# Why?

**Published:** 1897-04

**Authors:** W. L. Rhoads

**Affiliations:** Lansdowne, Pa.


By W. L. Rhoads, D.V.S.,

LANSDOWNE, PA.

Why, my fraternal brothers, you ask, have I chosen such a subject? "Why" allows me scope for a number of inquiries and assertions as the subject would of itself suggest.

"Why" are we veterinarians, as a class, prone to travel in our own individual route or rut? I use the latter term advisedly, for too many of us are at present living in a rut, or perhaps a myriad of ruts, which will be found very hard to leave when we are at last awakened by the onward movement of the Juggernaut car of science, which we will find overtaking us in the natural course of events unless we now take hold of the work before us and live according to the revised reading of the old axiom, "that all things come to him who waits." This, in accordance with the times, has added to it, "and hustles while he waits."

Are not periods of depression in our work times when we are taught to note how narrow has become our sphere of work and how great the field of our possibilities when properly managed? Lowell has said, "to a healthy mind the world is a challenge of opportunities."

"Why" not make these times of waiting hours of improvement, that we may more thoroughly grasp our whole duties; that we may be better able to cope with the difficulties and dangers that constantly beset us on every side?

[1] Read at the meeting of the Pennsylvania State Veterinary Medical Association, March 3, 1897.

We should remember that absence of occupation is not rest—"a mind quite vacant is a mind distressed." "Why" wait around aimlessly with your thumbs thrust into the armholes of your vest or possibly stuck deep into otherwise empty pockets? If you think you are posing, let me make a suggestion: don't continue in that line. Veterinarians, as a rule, make poor artist's models. About one in a thousand succeeds, and you are not the man. You stand just as good a chance for a foreign mission, and you cannot get that until all the good after-dinner speakers have declined.

"Why" not use this time in attending and assisting your local veterinary medical associations? "Why" not at least answer the invitation tendered you to do so? "Why" is it that not 3 per cent. of the veterinarians so honored are enough interested to answer the same? Is it due at this time to a rush of business or on account of the exorbitant price of postal cards? "Why" do you consider your fellow-practitioner less worthy of an immediate reply than any other correspondent? Are you interested in the profession only so far as it has a monetary value to you directly? If your interest is of a broader gauge, and I trust it is, why not show it by granting your fellow-practitioner an early reply to all correspondence? "Why" not show your interest in a more potent form by attending the meeting of your local veterinary medical association? If you are not a member, go as a visitor. You will always find the latch-string out, or, in later phraseology, "just press the button, and they will do the rest." You will soon become so interested that you will realize your need of association-fellowship and wonder how you existed without it. Or probably you are one of those who are willing to enshroud themselves in a mantle of egotism and feel the meetings of the association unworthy of their attendance and support. Yes, I meant to use the word shroud, as such an article will be useful in an early professional burial. Do you not realize that the rapid advancement of our profession is due in a great measure to our associations? Then why are you contented to continue your parasitic existence? You cannot even be considered as a sponge, for it will upon pressure or by slow evaporation give out all it has taken up.

"Why" not give to your fellow-practitioners at these meetings, or through the medium of the veterinary journals, more of your cases and experiences, that all may be benefited and our worth to our people enhanced all over the land? Some one may be encouraged and enthused by your effort to lighten the burden of the veterinarian, and in return may suggest the idea you have been groping after for years. "Why" not be willing at all times to give your professional brother a light from your cigar? He has gained; you have not lost; perhaps you have gained also by the aroma of his probably superior Havana, which had been useless to him until the procuring of your assistance. "Why" not be ever ready to reach out the right hand of good-fellowship and assist each other? Not willing to do it only on demand, but proffering it all times, thus showing to your professional brethren that you have an object in life—an object which in your case deservedly begins with a capital O.

What do we live for, if it is not to make life less difficult to others? "Why" not, in furtherance of this same end, take a greater interest in public affairs, and thus loan your valued influence to better government throughout the land? We, as a profession, take little appreciable interest in politics, and why? Is it because we are afraid of crossing our clientage? Can we not well afford to do without the practice of the narrow-minded cynic who ceases to employ us when we not only prove ourselves capable of administering to his stock, but prove we have a mind capable of independent thought by being men enough to assert the courage of our political convictions? "Why" is it that we find so few veterinarians on our local boards of health, when they are so well prepared both by education and training to act as sanitary experts in perfecting the healthfulness of the meat-, milk- and food-supply of a community as well as the sanitary surroundings?

"Why" is it that the veterinarians will vote, and in many cases assiduously work, to accomplish the desired ends for probably a valued client to us, financially, yet as a politician he may be a ringster and a spendthrift with the public moneys—moneys which too many of the legislators of to-day feel it a duty to spend in many cases wantonly and recklessly? Yet all this money comes through the channels of taxation from the individual who demands that the public receive it in a way which guarantees the greatest good to the greatest number. "Why" are our veterinarians assisting in this robbery of the populace by charging exorbitant fees for public work—fees which in many cases trebly repay them for that day's loss of private practice? Are you not putting a stumbling-block in the way of the furtherance of just such work? It is true a stumble may prevent a fall, but it is not well to stumble too often. It looks suspicious and may cause rumors; you will then have cause to ask yourself *Why?*

"Why" will our members deride and criticise legislation already gained (which, though probably not what we would like it to be, is yet a stepping-stone to better results), when they absolutely refuse to loan their assistance toward the procuring of this much-to-be-desired article? "Why" is it that men most loud in their protest against such legislation, and always ready to illustrate its faults, are the ones who positively would not lend their assistance in its preparation in order that it might have been free from the defects against which they cry out? "Why" will our members try to break down good laws that many of them have sorrowed over in the past because they did not exist? "Why" will members of the veterinary profession who believe in the merit system of appointment see it endangered for their own selfish ends when they know that for their own gratification, lasting but for a few short moments at best, they endanger a system which all aim to perpetuate in their own professional career?

"Why" not pause a moment to think? You will then realize that your efforts for self-aggrandizement at the expense of your fellow-men are of the boomerang character, and are apt to turn upon the projector. Such work will in the end receive such recompense as it merits.

"Those who by aspersions throw a stone at the heads of others hit their own."

"Why" will the young men of to-day deride and criticise the older ones in practice with whom they come in competition? They fail to realize that their position as practitioners has been made possible by these same men, who have worked shoulder-to-shoulder early and late when there was little apparent honor in it, at the same time breaking tidal wave after tidal wave of ignorance, arrogance, and depression, knowing full well that good intentions clothe themselves with sudden power.

"Why" is it that so many will be fully aware of many things punishable, yet apparently fail to see them till they begin to encroach upon forbidden personal territory? Then the cry of anguish is raised, and you are surprised that you are not immediately lifted out of a quagmire into which you persisted in going. "Why" not come out boldly at the first offence and at the first trampling upon your rights assert your manhood and professional standing, for right is right and wrongs no one? "Why" not go through life bearing the load which the contingency of the times has thrust upon us and bear it with honor—a credit to ourselves individually and to the profession as a whole? The time is not far distant when we as a profession will become the guiding-star of a whole nation. As sanitary humanitarians, are you in a condition or are you preparing yourself to share your portion of the burden? If not, why?

You are certainly not doing it by showing your preference for the literature contained in horse journals and agricultural magazines, when our professional ones contain so much that is not only beneficial in an educational way, but interesting not alone by its association but on account of its true worth. "Why" is it, in the face of this fact, that only one in every four North American veterinarians subscribes to and supports his own professional literature? It is true our country has been bordering on a panic which, thanks to the keen foresight and business-training of the American people, has been averted. Yet is that just cause for your making an auction mart of your profession? If not, why do you offer bargain-counter prices? Prices which you well know when once cut are so difficult to restore in times of prosperity? "Why" do veterinarians aim to establish prices for surgical work that are no just compensation for the skill required, and which cannot do otherwise than lessen the worth of the work in the eyes of laymen and the general public? "Why" will you take contract work at prices that are not half compensating, and thus lessen the volume of income to yourself individually and lower the estimate value of the work generally?

"Why" do veterinarians, many of whom have been educated at the supposedly better schools, feel that quackery is a paying branch of what would otherwise be a wholly honorable profession? "Why" do they feel that placards bearing pictures and self-laudations, wholly unprofessional in appearance and work, when they only tend to lower them in the public estimation? "Why" do we not impress upon the dairy people of this State the urgent need of stringent police measures and the enforcement of the same, that they may know all cattle brought into the State to replenish their herds are free from all infectious and contagious disease, when the losses already incurred from this source are assuming a serious aspect as regards the dairying interests, and threaten to become a barrier to the continuance of the valuable control-work now going on—work which at this time must not be stopped, and which can be best perpetuated by the veterinarians individually assisting it, for we live in deeds, not years; in thoughts, not breaths; in feelings, not in figures on a dial?

